# The SCF-FBW7β E3 ligase mediates ubiquitination and degradation of the serine/threonine protein kinase PINK1

**DOI:** 10.1016/j.jbc.2024.107198

**Published:** 2024-03-18

**Authors:** Seo Jeong Jeon, Kwang Chul Chung

**Affiliations:** Department of Systems Biology, College of Life Science and Biotechnology, Yonsei University, Seoul, South Korea

**Keywords:** PINK1, FBW7, SCF complex, ubiquitination, proteasomal degradation, neurodegenerative diseases, Parkinson's disease

## Abstract

Understanding the mechanisms that govern the stability of functionally crucial proteins is essential for various cellular processes, development, and overall cell viability. Disturbances in protein homeostasis are linked to the pathogenesis of neurodegenerative diseases. PTEN-induced kinase 1 (PINK1), a protein kinase, plays a significant role in mitochondrial quality control and cellular stress response, and its mutated forms lead to early-onset Parkinson's disease. Despite its importance, the specific mechanisms regulating PINK1 protein stability have remained unclear. This study reveals a cytoplasmic interaction between PINK1 and F-box and WD repeat domain–containing 7β (FBW7β) in mammalian cells. FBW7β, a component of the Skp1-Cullin-1-F-box protein complex–type ubiquitin ligase, is instrumental in recognizing substrates. Our findings demonstrate that FBW7β regulates PINK1 stability through the Skp1-Cullin-1-F-box protein complex and the proteasome pathway. It facilitates the K48-linked polyubiquitination of PINK1, marking it for degradation. When FBW7 is absent, PINK1 accumulates, leading to heightened mitophagy triggered by carbonyl cyanide 3-chlorophenylhydrazone treatment. Moreover, exposure to the toxic compound staurosporine accelerates PINK1 degradation *via* FBW7β, correlating with increased cell death. This study unravels the intricate mechanisms controlling PINK1 protein stability and sheds light on the novel role of FBW7β. These findings deepen our understanding of PINK1-related pathologies and potentially pave the way for therapeutic interventions.

Parkinson's disease (PD) is a complex neurodegenerative disorder marked by the gradual degeneration of dopaminergic neurons in the *substantia nigra pars compacta*, resulting in motor and nonmotor symptoms (1). Although the exact causes of PD remain elusive, a growing body of evidence indicates that both genetic and environmental factors contribute to its development (2). Among the genes linked to familial PD, PTEN-induced kinase 1 (PINK1) has emerged as a vital player in mitochondrial quality control and cellular stress response (3, 4, 5, 6). PINK1, primarily localized in the mitochondria, plays a crucial role in maintaining mitochondrial integrity and function as a serine/threonine kinase (6). It acts as a sensor for mitochondrial damage and orchestrates the recruitment of Parkin, another PD-associated protein, to damaged mitochondria, leading to their removal through a process known as mitophagy (4, 5, 6). Loss-of-function mutations in PINK1 disrupt the mitophagic process, causing the accumulation of damaged mitochondria, increased oxidative stress, and neuronal degeneration in PD (4). While the significant role of PINK1 in PD pathogenesis is well-established, the precise mechanisms governing its regulation and protein stability are not fully understood yet.

Elucidating the regulation of PINK1 stability is crucial for comprehending PD pathology because PINK1 is involved in a prosurvival pathway. The ubiquitin-proteasome system (UPS) plays a role in regulating PINK1 stability. For example, the stabilization of PINK1 on damaged mitochondria depends on TNF receptor-associated factor 6 (TRAF6)-mediated K63-linked ubiquitination of PINK1 (7). Additionally, cleaved PINK1 undergoes rapid turnover, recognized by N-end rule E3 enzymes, facilitating the identification and elimination of damaged mitochondria through autophagy (8). Until recently, proteasomal degradation mediated by the N-end rule was the sole known pathway for PINK1 turnover, limited to the cleaved form of PINK1 (8). However, our recent discovery revealed that full-length PINK1 can also undergo proteasomal degradation mediated by the carboxyl terminus of Hsp70-interacting protein (CHIP) E3 ligase (9).

The F-box and WD repeat domain–containing 7 (FBW7), also known as FBXW7, CDC4, and Sel-10, functions as an E3 ubiquitin ligase, playing a pivotal role in protein degradation *via* the ubiquitin-proteasome system (10, 11, 12). FBW7 comprises various functional domains, including an F-box domain facilitating protein–protein interactions and WD40 repeats contributing to substrate recognition (13, 14, 15). Similar to other F-box–containing proteins, FBW7 may be part of the Skp1-Cullin-1-F-box protein (SCF) complex, recognizing and binding target substrates through the CDC4-phosphodegron motif (16, 17, 18, 19). FBW7 exists in three major isoforms: FBW7α, FBW7β, and FBW7γ, resulting from alternative splicing of the *FBW7* gene. These isoforms exhibit differences in tissue distribution and substrate specificity (11, 12, 20). Notably, they differ in cellular localization due to N-terminal variations, with α predominantly in the nucleus, β in the cytoplasm, and γ in the nucleolus (10, 11, 12, 20). While these isoforms share the F-box domain and substrate recognition motifs, the distinct N-terminal region theoretically allows all three isoforms to recognize a wide array of substrates, rendering them functionally identical (10, 20).

FBW7 is well known for its involvement in the degradation of several oncogenic proteins, including cyclin E, c-Myc, Notch, and c-Jun (19, 21, 22). By targeting these proteins, FBW7 acts as a tumor suppressor, crucial for maintaining cellular homeostasis (10, 11, 12, 21, 22). Additionally, FBW7 has been linked to various human diseases, including cancers, neurological disorders, and cardiovascular diseases (21, 22, 23). In the context of neurological disorders, FBW7 has garnered attention for its potential role in neurodevelopmental disorders and neurodegenerative diseases such as Alzheimer's disease (AD) and PD (23). Dysregulation of FBW7-mediated protein degradation pathways could contribute to the accumulation of pathological proteins and the development of neurodegeneration (23).

While the enzymes involved in the degradation of PINK1 are limited to ubiquitin protein ligase E3 component N-recognin (UBR) and CHIP, it is widely acknowledged that protein ubiquitination can be intricately regulated by multiple E3 ligases (8, 9, 24). This study aims to explore whether FBW7β serves as a novel E3 ligase for PINK1 and, if so, to further investigate its impact on mitophagy and cell death in the context of PINK1 degradation. Our research demonstrates the functional relationship between PINK1 and FBW7 and establishes that FBW7β acts as a key regulator of PINK1 stability through the SCF complex and the subsequent UPS pathway. These findings contribute in understanding the regulatory mechanisms governing PINK1 stability, which is essential for unraveling the pathogenic processes in PD and identifying potential therapeutic targets.

## Results

### PINK1 interacts with FBW7β in mammalian cells

**Figure 1:**
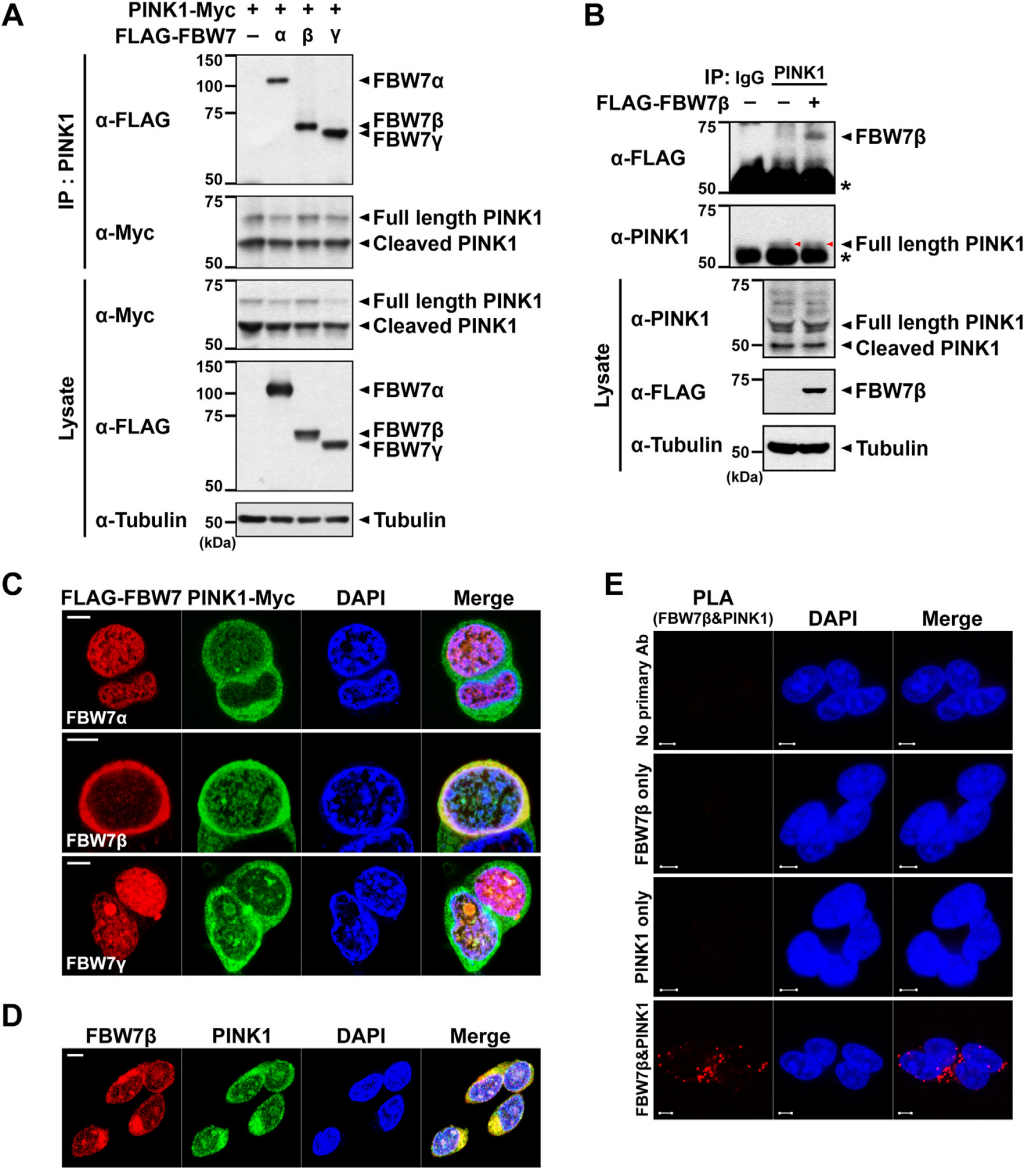
**PINK1 is a binding partner of FBW7β.***A*, HEK293 cells were transfected with plasmids encoding PINK1-Myc alone or together with FLAG-FBW7α, FLAG-FBW7β, or FLAG-FBW7γ for 24 h. Total cell lysates were immunoprecipitated with anti-PINK1 antibody, followed by immunoblotting with the indicated antibodies. Tubulin served as a loading control. *B*, where indicated, SH-SY5Y cells were mock-transfected (−) or transfected with a plasmid encoding FLAG-FBW7β for 48 h, followed by treatment with 20 μM MG132 for 4 h before harvesting. Cell lysates were immunoprecipitated with anti-PINK1 antibody, followed by immunoblotting with the indicated antibodies. Preimmune IgG was used as a negative control for immunoprecipitation. Tubulin served as a loading control. The *asterisk* indicates IgG heavy chains. The bands marked with a *red arrowhead* indicated the PINK1 bands. *C*, where indicated, HEK293 cells were cotransfected with plasmids encoding PINK1-Myc and one of the FLAG-FBW7α, FLAG-FBW7β, or FLAG-FBW7γ for 24 h. Representative confocal images of immunostained ectopic FBW7 isoforms and PINK1 are shown. The scale bar represents 5 μm. *D*, the representative confocal images of the colocalization of endogenous FBW7β (*red*) and PINK1 (*green*) in SH-SY5Y cells are shown. The scale bar represents 5 μm. *E*, PLAs were performed using primary antibodies against PINK1 and FBW7β. Representative PLA images (*red*) depicting the interaction between endogenous PINK1 and FBW7β are shown. The scale bar represents 5 μm. FBW7, F-box and WD repeat domain–containing 7; PINK1, PTEN-induced kinase 1; PLA, proximity ligation assay.

In this study, we explored the interactions between PINK1 and different FBW7 isoforms in mammalian cells. HEK293 cells were transfected with plasmids encoding Myc-tagged PINK1 and FLAG-tagged FBW7α, FBW7β, or FBW7γ isoform. Coimmunoprecipitation (co-IP) analysis of cell lysates demonstrated that overexpressed PINK1 interacted with all three FBW7 isoforms (Fig. 1*A*). Considering the shared domains among FBW7 isoforms and their distinct subcellular localizations primarily attributed to N-terminal differences (10, 11, 12, 20), we further examined the colocalization of PINK1 with these isoforms. Fluorescence microscopic analysis of HEK293 cells transfected with PINK1-Myc and one of the FLAG-tagged FBW7 isoforms revealed that while FBW7β predominantly localized in the cytosol, FBW7α and FBW7γ isoforms were confined to the nucleus. Notably, cytosolic PINK1 colocalized specifically with FBW7β, not with FBW7α or FBW7γ (Fig. 1*C*). This observation was corroborated using fluorescence microscopy analysis with endogenous antibodies against PINK1 and FBW7β (Fig. 1*D*).

To further confirm the interaction between ectopic FBW7β and endogenous PINK1, co-IP analysis was performed. Given the lack of commercially available antibodies for detecting endogenous FBW7β effectively in Western blotting, FLAG-tagged FBW7β was transiently overexpressed in SH-SY5Y cells. The co-IP assay validated the binding of endogenous PINK1 with ectopic FBW7β (Fig. 1*B*). To specifically validate their interaction, the proximity ligation assay (PLA) was utilized to visualize their predominant interaction regions within SH-SY5Y cells. Consistent with previous findings, PLA revealed that endogenous PINK1 and FBW7β primarily interacted in the cytosol (Fig. 1*E*). Collectively, these findings provide strong evidence supporting the specific binding between PINK1 and FBW7β in mammalian cells.

### FBW7β negatively regulates the protein stability of PINK1

**Figure 2:**
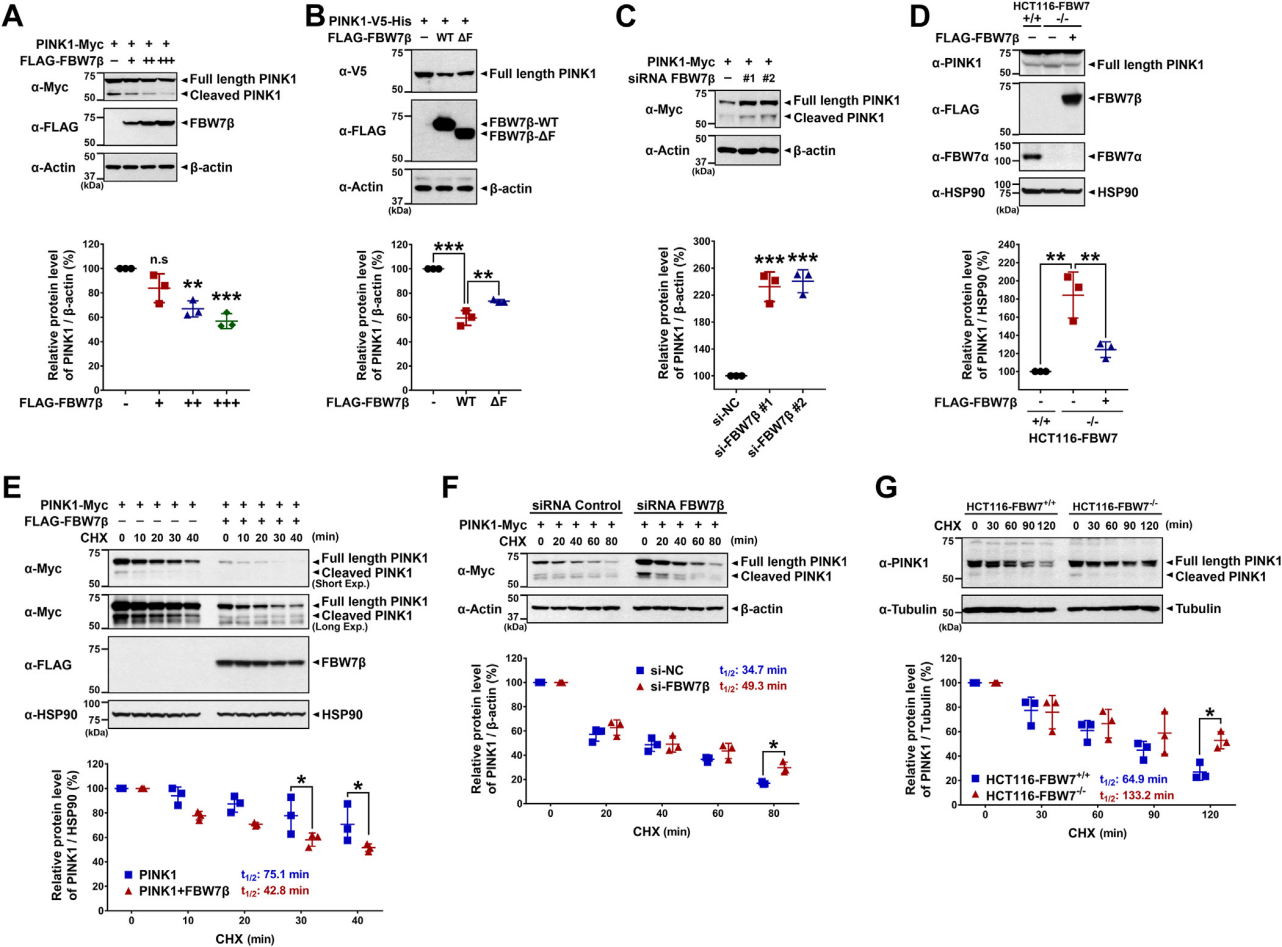
**FBW7β negatively regulates the protein stability of PINK1.***A*, where indicated, HEK293 cells were transfected for 24 h with plasmids encoding PINK1-Myc alone or together with increasing doses of FLAG-FBW7β-WT. Whole-cell lysates were subsequently immunoblotted with the indicated antibodies. Relative PINK1 levels compared to β-actin were quantified, and the data are presented as the mean ± SEM of three independent experiments (∗∗*p* < 0.01; ∗∗∗*p* < 0.001; n.s, not significant). *B*, HEK293 cells were transfected for 24 h with plasmids encoding PINK1-Myc alone or together with either FLAG-FBW7β-WT or FLAG-FBW7β-ΔF. Whole-cell lysates were subsequently immunoblotted with the indicated antibodies. Relative PINK1 levels compared to β-actin were quantified, and the data are presented as the mean ± SEM of three independent experiments (∗∗*p* < 0.01; ∗∗∗*p* < 0.001). *C*, HEK293 cells were transfected for 48 h with plasmids encoding PINK1-Myc, nonspecific scrambled control siRNA, or *FBW7β*-siRNA alone or in combination. Cell lysates were immunoblotted with the indicated antibodies. Relative PINK1 levels compared to β-actin were quantified, and the data are presented as the mean ± SEM of three independent experiments (∗∗∗*p* < 0.001). *D*, the FBW7^+/+^ and FBW7^−/−^ HCT116 cells were mock-transfected or transfected with a plasmid encoding FLAG-FBW7β for 48 h. Whole-cell lysates were immunoblotted with the indicated antibodies. Relative PINK1 levels compared to HSP90 were quantified, and the data are presented as the mean ± SEM of three independent experiments (∗∗*p* < 0.01). *E*, HEK293 cells were transfected for 24 h with plasmids encoding PINK1-Myc alone or together with FLAG-FBW7β. Cells were then treated with 50 μg/ml cycloheximide (CHX) for the indicated times, and cell lysates were immunoblotted with the indicated antibodies. Relative PINK1 levels compared to HSP90 were quantified, and the data are presented as the mean ± SEM of three independent experiments (∗*p* < 0.05). *F*, HEK293 cells were transfected for 48 h with scrambled control siRNA, *FBW7β*-siRNA, or plasmid encoding Myc-tagged PINK1 alone or in combination. Cells were then treated with 50 μg/ml CHX for the indicated times, and cell lysates were immunoblotted with the indicated antibodies. Relative PINK1 levels compared to β-actin were quantified, and the data are presented as the mean ± SEM of three independent experiments (∗*p* < 0.05). *G*, the FBW7^+/+^ and FBW7^−/−^ HCT116 cells were treated with 50 μg/ml CHX for the indicated times, and cell lysates were immunoblotted with the indicated antibodies. Relative PINK1 levels compared to tubulin were quantified, and the data are presented as the mean ± SEM of three independent experiments (∗*p* < 0.05). Tubulin, β-actin, and HSP90 were used as a control for equal protein loading. FBW7, F-box and WD repeat domain–containing 7; PINK1, PTEN-induced kinase 1.

FBW7 functions as part of an SCF complex and operates as an E3 ligase, orchestrating the ubiquitination and subsequent degradation of its target substrates (13, 14, 15). To gain further insight as to how FBW7β and PINK1 are linked, we investigated whether FBW7β influences the protein stability of PINK1. Initial Western blotting experiments revealed a gradual decrease in the level of PINK1-Myc upon the dose-dependent introduction of FLAG-FBW7β (Fig. 2*A*). Intriguingly, this effect was absent in the presence of a FBW7β mutant lacking the F-box domain (FBW7β-ΔF), which is unable to recognize target substrates and consequently had no impact on the level of PINK1-Myc (Fig. 2*B*). Additionally, we explored the effect of siRNA-mediated knockdown of FBW7β on PINK1-Myc levels. As a control, we verified the efficiency of *FBW7β* knockdown by comparing FBW7β mRNA levels through real-time quantitative PCR analyses (Fig. S1*A*). Contrary to the impact of FBW7β overexpression, suppressing FBW7β led to an increase in PINK1-Myc levels (Fig. 2*C*).

Furthermore, we examined the change in endogenous PINK1 levels in the human colon cancer HCT116 cell line with *FBW7* deletion. Compared to the PINK1 level in WT FBW7-expressing cells (used as a control), *FBW7*-null cells exhibited a significant increase, which was subsequently reduced upon the overexpression of FLAG-FBW7β in the *FBW7*-KO cells (Fig. 2*D*). To confirm the successful knockout of *FBW7* in HCT116 cells, we used an endogenous anti-FBW7α antibody due to issues related to specific FBW7β antibodies. Additionally, real-time quantitative PCR analyses were conducted (Figs. 2*D* and S1*B*). Both assays verified the complete and efficient blockage of FBW7α and β isoform expressions in the HCT116 cells, respectively.

Subsequently, we determined how FBW7β impacts the half-life of PINK1. Overexpression of FLAG-FBW7β led to a decrease in the half-life of PINK1-Myc (Fig. 2*E*), while FBW7β knockdown, using *FBW7β*-siRNA, resulted in an increased PINK1 half-life (Fig. 2*F*). Similarly, compared to the WT FBW7 HCT116 cell line, an increased half-life of endogenous PINK1 was observed in *FBW7*-KO cells (Fig. 2*G*). These results collectively indicate that FBW7β negatively regulates the protein stability of PINK1.

### FBW7β facilitates the degradation of PINK1 through the SCF complex–dependent and proteasome pathway

**Figure 3:**
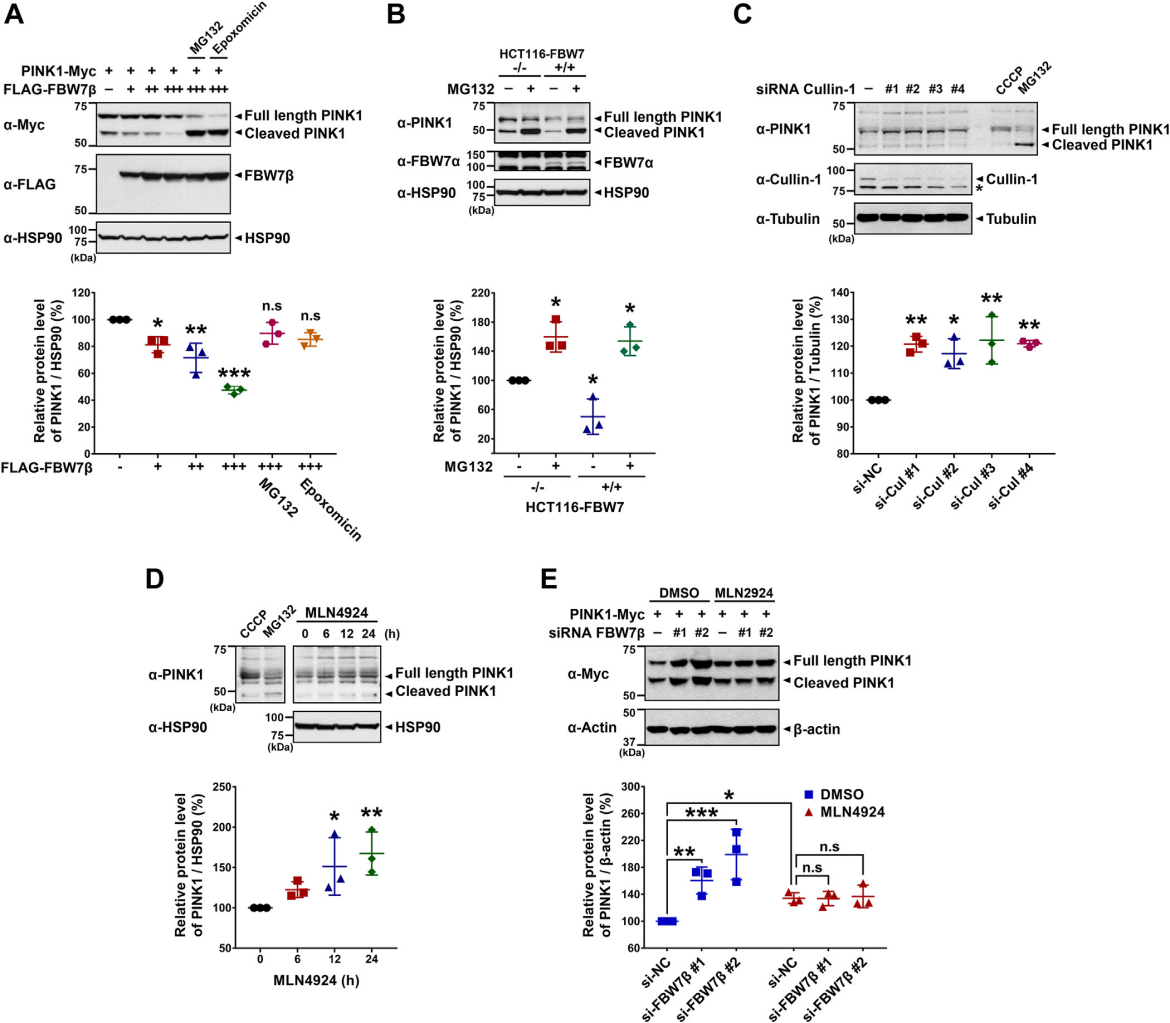
**FBW7β promotes the degradation of PINK1 *via* the SCF complex–dependent proteasome pathway.***A*, HEK293 cells were transfected with plasmid encoding PINK1-Myc or FLAG-FBW7β alone or in combination for 24 h. Cells were then treated with vehicle, 20 μM MG132, or 2 μM epoxomicin for 6 h before harvesting. Cell lysates were subjected to immunoblotting with the indicated antibodies. The relative PINK1 levels compared to HSP90 were quantified. The presented data represent the mean ± SEM of three independent experiments (∗*p* < 0.05; ∗∗*p* < 0.01; ∗∗∗*p* < 0.001; n.s., not significant). *B*, after FBW7^+/+^ and FBW7^−/−^ HCT116 cells were treated with vehicle or 10 μM MG132 for 6 h, cell lysates were immunoblotted with the indicated antibodies. The relative PINK1 levels compared to HSP90 were quantified. The presented data represent the mean ± SEM of three independent experiments (∗*p* < 0.05). *C*, where indicated, HEK293 cells were transfected with scrambled control siRNA or *cullin-1*-siRNA for 48 h, and treated with 20 μM MG132 or 20 μM CCCP for additional 4 h. Cell lysates were immunoblotted with the indicated antibodies. The relative PINK1 levels compared to tubulin were quantified. The presented data represent the mean ± SEM of three independent experiments (∗*p* < 0.05; ∗∗*p* < 0.01). *D*, SH-SY5Y cells were treated with 1 μM MLN4924 for the indicated times and treated with 20 μM MG132 or 20 μM CCCP for additional 6 h. Cell lysates were immunoblotted with the indicated antibodies. The relative PINK1 levels compared to HSP90 were quantified. The presented data represent the mean ± SEM of three independent experiments (∗*p* < 0.05; ∗∗*p* < 0.01). The CCCP treatment was employed to denote the size of endogenous full-length PINK1 band, while MG132 treatment was used to represent the size of endogenous cleaved PINK1 band. *E*, HEK293 cells were transfected with scrambled control siRNA, *FBW7β*-siRNA, or plasmid encoding PINK1-Myc alone or in combination for 48 h, and treated with vehicle or 2 μM MLN4924 for additional 24 h. Cell lysates were immunoblotted with the indicated antibodies. The relative PINK1 levels compared to β-actin were quantified. The presented data represent the mean ± SEM of three independent experiments (∗*p* < 0.05; ∗∗*p* < 0.01; ∗∗∗*p* < 0.001; n.s., not significant). Tubulin, β-actin, and HSP90 were used as a control for equal protein loading. CCCP, carbonyl cyanide 3-chlorophenylhydrazone; FBW7, F-box and WD repeat domain–containing 7; PINK1, PTEN-induced kinase 1; SCF, Skp1-Cullin-1-F-box protein.

We then investigated whether the downregulation of PINK1 by FBW7β occurs through the SCF complex– and/or UPS-dependent degradation pathway. In Figure 3*A*, HEK293 cells were transfected with plasmids encoding PINK1-Myc alone or cotransfected with increasing doses of FLAG-FBW7β and subsequently treated with proteasome inhibitors, including MG132 or epoxomicin. Western blot analysis using anti-Myc antiserum revealed a gradual dose-dependent decrease in PINK1-Myc levels in the presence of FLAG-FBW7β, which was subsequently restored upon treatment with the proteasome inhibitor (Fig. 3*A*). To further validate this, *FBW7*-KO HCT116 cells and control cell lines were treated with MG132 and endogenous PINK1 levels were compared. Western blot analysis with anti-PINK1 antibodies demonstrated a significantly higher accumulation of endogenous PINK1 in *FBW7*-null cells than WT cells. Treatment with MG132 led to an increase in PINK1 levels in both WT and *FBW7*-KO cells (Fig. 3*B*).

Additionally, we investigated whether the proteolytic function of FBW7β primarily stemmed from its E3 ligase activity within the SCF complex. By blocking the expression of cullin-1, a crucial component of the SCF complex, using *cullin-1*-siRNA, we examined its effect on endogenous PINK1 levels. Compared with control cells transfected with scrambled control siRNA, cells treated with all four types of *cullin-1*-siRNA displayed increased endogenous PINK1 levels (Fig. 3*C*). Furthermore, to modulate the enzymatic activity of the SCF complex, cells were treated with MLN4924, a small molecule inhibitor that effectively inactivates SCF E3 by blocking cullin-1 neddylation (25, 26). When SH-SY5Y cells were exposed to MLN4924, a gradual, time-dependent increase in endogenous PINK1 levels was observed (Fig. 3*D*). Moreover, knockdown of FBW7β expression using *FBW7β*-siRNA resulted in elevated PINK1-Myc levels, while treatment with MLN4924 did not induce significant alterations in PINK1 levels (Fig. 3*E*).

In summary, these findings suggest that FBW7β promotes the degradation of PINK1 through the SCF complex–dependent and proteasome pathways.

### FBW7β facilitates K48-linked polyubiquitination of PINK1

**Figure 4:**
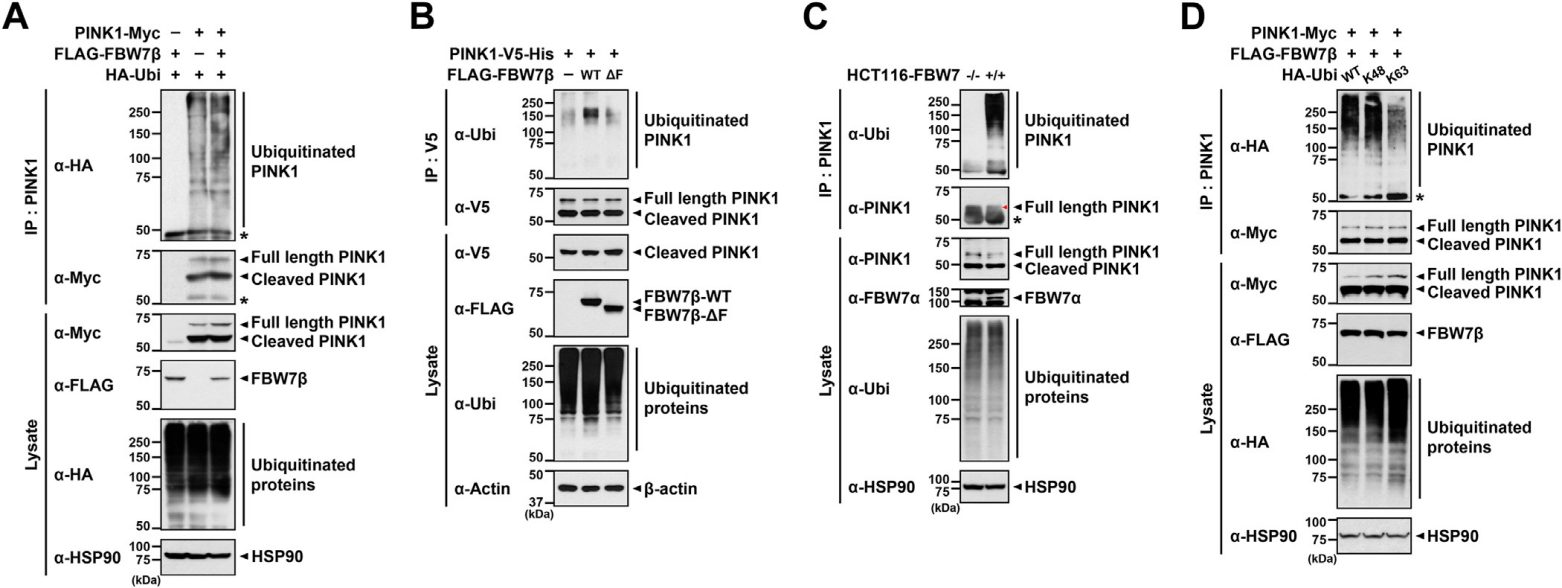
**FBW7β promotes K48-linked polyubiquitination of PINK1.***A*, HEK293 cells were transfected with plasmids encoding PINK1-Myc, FLAG-FBW7β, or HA-tagged ubiquitin (HA-Ubi) alone or in combination for 24 h, and treated with 20 μM MG132 for additional 4 h. Cell lysates were immunoprecipitated with anti-PINK1 antibody, followed by immunoblotting with the indicated antibodies. *B*, HEK293 cells were transfected with plasmids encoding PINK1-V5-His, FLAG-FBW7β-WT, or FLAG-FBW7β-ΔF alone or in combination for 24 h, and incubated with 20 μM MG132 for an additional 4 h. Cell lysates were immunoprecipitated with anti-V5 antibody, followed by immunoblotting with the indicated antibodies. *C*, FBW7^+/+^ and FBW7^−/−^ HCT116 cells were treated with 10 μM MG132 for 6 h. Immunoprecipitation (IP) of cell lysates was performed using an anti-PINK1 antibody, followed by Western blotting with anti-ubiquitin (Ubi) or anti-PINK1 antibody. The *red arrowheads* indicated the PINK1 bands. *D*, HEK293 cells were transfected with plasmids encoding PINK1-Myc, FLAG-FBW7β, HA-Ubi-WT, or one of two HA-Ubi mutants where the Lys residues except for the numbered one (K48, K63) were replaced with Arg either alone or in combination for 24 h. Cells were then treated with 20 μM MG132 for additional 4 h. Cell lysates were immunoprecipitated with anti-PINK1 antibody, followed by immunoblotting with the specified antibodies. β-Actin and HSP90 were used as controls for equal protein loading. The *asterisk* indicates IgG heavy chains. FBW7, F-box and WD repeat domain–containing 7; HA, hemagglutinin; PINK1, PTEN-induced kinase 1.

To explore the role of FBW7β in PINK1 ubiquitination, HEK293 cells were transfected with plasmids encoding PINK1-Myc, FLAG-FBW7β, or HA-ubiquitin alone or in combination. Co-IP analysis of cell lysates using anti-PINK1 antibody, followed by immunoblotting with anti-HA antibody revealed an increase in the polyubiquitination of Myc-tagged PINK1 upon FLAG-FBW7β overexpression (Fig. 4*A*). Conversely, transfection with the F-box domain–lacking mutant of FLAG-FBW7β (FBW7β-ΔF), which impairs its ability to recognize target substrates, showed no significant change in PINK1 ubiquitination compared to the WT FBW7β control (Fig. 4*B*). Additionally, comparison of PINK1 ubiquitination levels in *FBW7*-null HCT116 and its control cell lines demonstrated a notable decrease in endogenous PINK1 ubiquitination in *FBW7*-null cells (Fig. 4*C*). Given the previous knowledge that FBW7-mediated ubiquitination predominantly involves K48-linked ubiquitin chains (27), we examined whether FBW7β enhances PINK1 ubiquitination through K48- or K63-linked ubiquitin chains. As illustrated in Fig. 4*D*, FBW7β significantly promoted PINK1 ubiquitination predominantly through K48-linked chains rather than K63 linkage. Since K48-linked ubiquitin chains target proteins for proteasomal degradation, while K63-linked chains do not, these results support the notion that FBW7β promotes PINK1 degradation through the proteasome pathway.

In summary, these findings indicate that FBW7β promotes K48-linked polyubiquitination of PINK1.

### Depletion of FBW7β enhances carbonyl cyanide 3-chlorophenylhydrazone–induced mitophagy through the accumulation of PINK1

**Figure 5:**
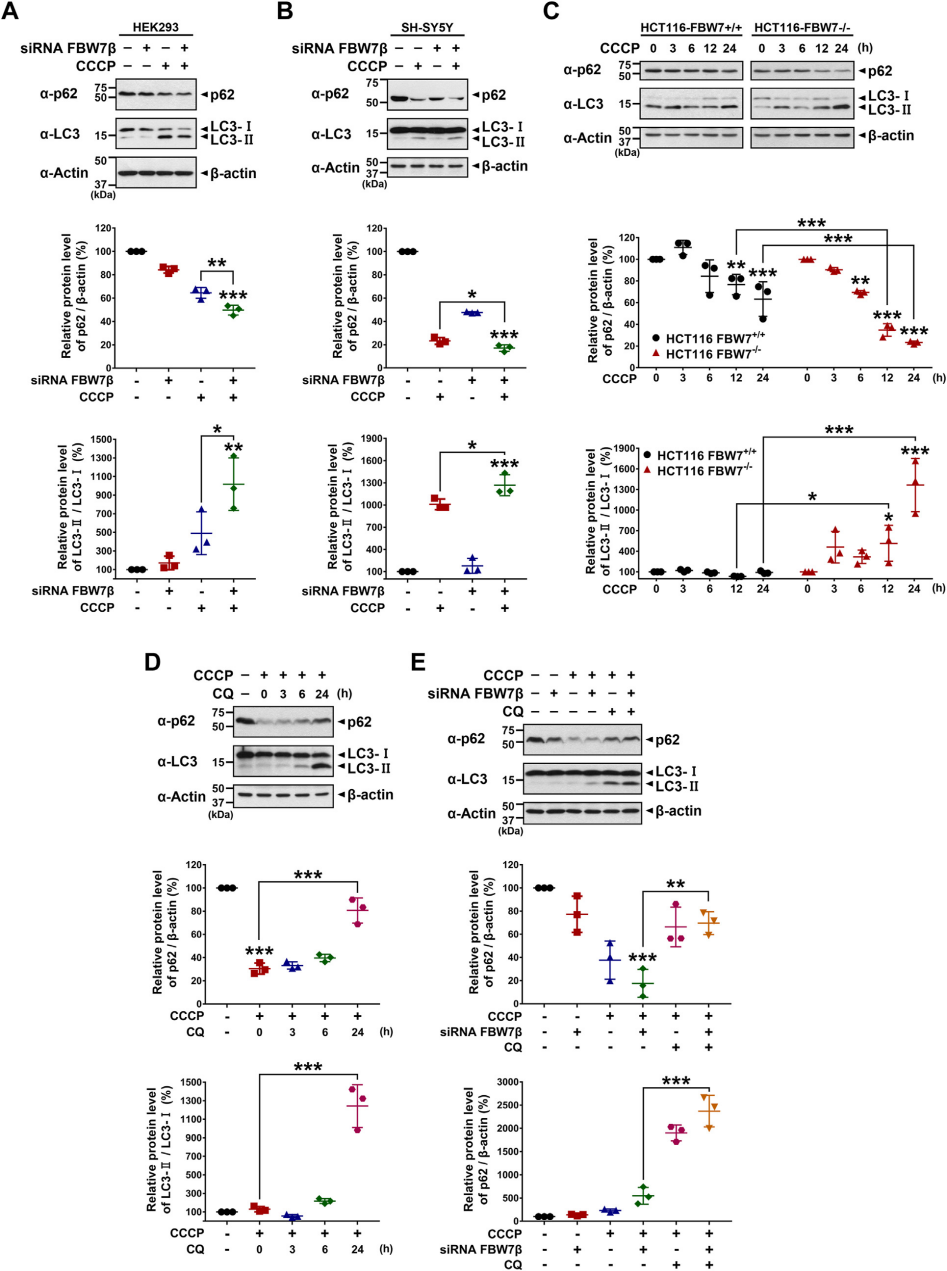
**FBW7β depletion regulates the levels of autophagy markers, increasing LC3 and decreasing p62, upon CCCP treatment.***A*, HEK293 cells were transfected with scrambled control siRNA or *FBW7β*-siRNA for 48 h and treated with vehicle or 10 μM CCCP for additional 24 h. Cell lysates were immunoblotted with the indicated antibodies. The relative p62 levels compared to β-actin and the relative LC3-Ⅱ levels compared to LC3-Ⅰ were quantified. The presented data represent the mean ± SEM of three independent experiments (∗*p* < 0.05; ∗∗*p* < 0.01; ∗∗∗*p* < 0.001). *B*, SH-SY5Y cells were transfected with scrambled control siRNA or *FBW7β*-siRNA for 48 h and treated with vehicle or 10 μM CCCP for additional 24 h. Cell lysates were immunoblotted with the indicated antibodies. The relative p62 levels compared to β-actin and the relative LC3-Ⅱ levels compared to LC3-Ⅰ were quantified. The presented data represent the mean ± SEM of three independent experiments (∗*p* < 0.05; ∗∗∗*p* < 0.001). *C*, FBW7^+/+^ and FBW7^−/−^ HCT116 cells were treated with vehicle or 10 μM CCCP for the indicated times. Cell lysates were immunoblotted with the specified antibodies. The relative p62 levels compared to β-actin and the relative LC3-Ⅱ levels compared to LC3-Ⅰ were quantified. The presented data represent the mean ± SEM of three independent experiments (∗*p* < 0.05; ∗∗*p* < 0.01; ∗∗∗*p* < 0.001). *D*, SH-SY5Y cells were treated with vehicle or 10 μM CCCP for 24 h and then treated with vehicle or 50 μM CQ for the indicated times. Cell lysates were immunoblotted with the indicated antibodies. The relative p62 levels compared to β-actin and the relative LC3-Ⅱ levels compared to LC3-Ⅰ were quantified. The presented data represent the mean ± SEM of three independent experiments (∗∗∗*p* < 0.001). *E*, SH-SY5Y cells were transfected with scrambled control siRNA or *FBW7β*-siRNA for 48 h and treated with 10 μM CCCP alone or in combination with 50 μM CQ for additional 24 h. The cell lysates were immunoblotted with the indicated antibodies. The relative p62 levels compared to β-actin and the relative LC3-Ⅱ levels compared to LC3-Ⅰ were quantified. The presented data represent the mean ± SEM of three independent experiments (∗∗*p* < 0.01; ∗∗∗*p* < 0.001). FBW7, F-box and WD repeat domain–containing 7; PINK1, PTEN-induced kinase 1; CCCP, carbonyl cyanide 3-chlorophenylhydrazone; CQ, chloroquine.

We further investigated whether the degradation of PINK1 *via* the action of FBW7β has an impact on the mitophagy within cells. Initially, we monitored the alterations in the autophagy marker proteins, such as p62 and LC3-II form. We first knocked down the *FBW7β* expression in HEK293 and SH-SY5Y cells using siRNA and treated them with carbonyl cyanide 3-chlorophenylhydrazone (CCCP), a well-established mitophagy inducer. As a result, we observed a significant decrease in the level of p62 in the samples where FBW7β was knocked down and CCCP was treated, while an increase in the conversion from LC3-I to LC3-II form was noted (Fig. 5, *A* and *B*). Subsequently, we treated *FBW7*-KO or control HCT116 cells with CCCP over time and observed changes in protein levels. Consistently, a pronounced decrease in the level of p62 was evident in FBW7^−/−^ cells compared to FBW7^+/+^ cells, whereas there was an increasing trend observed in the LC3-II form (Fig. 5*C*). Furthermore, we investigated whether the altered levels were restored upon treatment with chloroquine (CQ), a widely recognized autophagy inhibitor known for its ability to increase lysosomal pH and impede autophagy flux. After cells were consecutively treated with CCCP and CQ over time, there was a gradual increase in the level of p62, which had been decreased due to CCCP (Fig. 5*D*). Conversely, the level of LC3-II, which had been increased following CCCP treatment, exhibited further accumulation upon treatment with CQ, likely due to the inhibition of autophagy flux by CQ hindering the fusion of autophagosomes with lysosomes, leading to the impaired degradation of LC3-II (Fig. 5*D*). Lastly, after SH-SY5Y cells were treated with *FBW7β*-siRNA and CCCP, followed by CQ treatment, we measured the protein level changes. Notably, we observed the restoration of p62 levels, which had been significantly reduced in the absence of FBW7 and following CCCP treatment, upon administration of CQ (Fig. 5*E*). Moreover, although the conversion from LC3-I to LC3-II was notably enhanced in the absence of FBW7 and following CCCP treatment, we observed additional accumulation of LC3-II upon CQ treatment (Fig. 5*E*). These findings underscore the intricate regulatory role of FBW7 in modulating the autophagic process.

**Figure 6:**
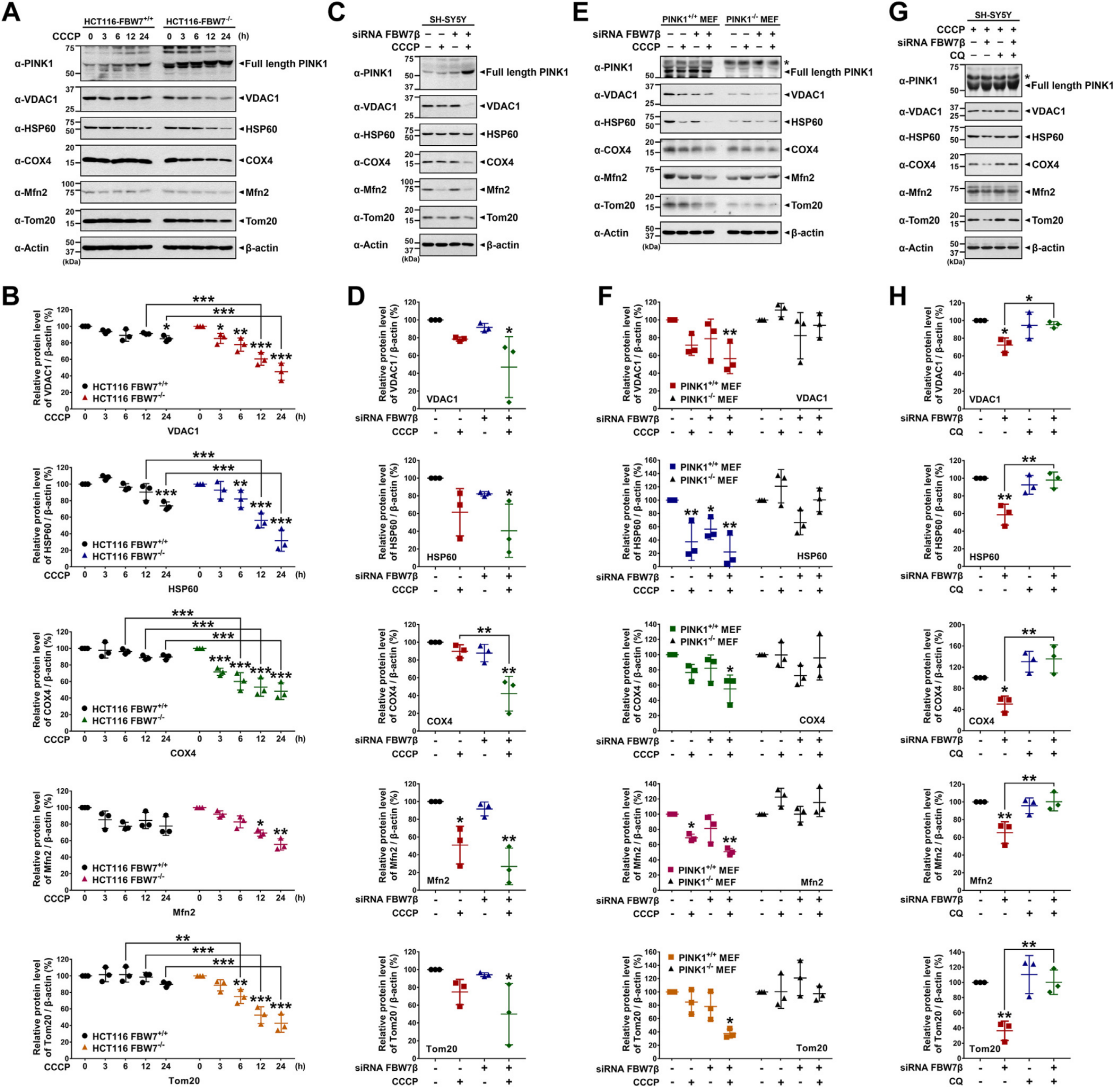
**FBW7β depletion promotes reduction in mitochondrial proteins upon CCCP treatment.***A*, FBW7^+/+^ and FBW7^−/−^ HCT116 cells were treated with vehicle or 10 μM CCCP for the indicated times. Cell lysates were immunoblotted with the specified antibodies. β-Actin was used as a control for equal protein loading. *B*, relative levels of five mitochondrial proteins (*i.e.,* VDAC1, HSP60, COX4, Mfn2, and Tom20) compared with β-actin were quantified, and the data are presented as the mean ± SEM of three independent experiments (∗*p* < 0.05; ∗∗*p* < 0.01; ∗∗∗*p* < 0.001). *C*, SH-SY5Y cells were transfected for 48 h with scrambled control siRNA or *FBW7β*-siRNA and treated with vehicle or 10 μM CCCP for additional 24 h. The cell lysates were immunoblotted with the specified antibodies. β-Actin was used as a control for equal protein loading. *D*, relative levels of five mitochondrial proteins (*i.e.,* VDAC1, HSP60, COX4, Mfn2, and Tom20) compared with β-actin were quantified, and the data are presented as the mean ± SEM of three independent experiments (∗*p* < 0.05; ∗∗*p* < 0.01). *E*, PINK1^+/+^ and PINK1^−/−^ MEF cells were transfected for 48 h with scrambled control siRNA or *FBW7β*-siRNA and treated with vehicle or 10 μM CCCP for additional 24 h. The cell lysates were immunoblotted with the indicated antibodies. The *asterisk* indicates nonspecific bands. *F*, relative levels of five mitochondrial proteins (*i.e.,* VDAC1, HSP60, COX4, Mfn2, and Tom20) compared with β-actin were quantified, and the data are presented as the mean ± SEM of three independent experiments (∗*p* < 0.05; ∗∗*p* < 0.01). *G*, SH-SY5Y cells were transfected with scrambled control siRNA or *FBW7β*-siRNA for 48 h and treated with 10 μM CCCP alone or in combination with 50 μM CQ for additional 24 h. The cell lysates were immunoblotted with the indicated antibodies. The *asterisk* indicates nonspecific bands. *H*, relative levels of five mitochondrial proteins (*i.e.,* VDAC1, HSP60, COX4, Mfn2, and Tom20) compared with β-actin were quantified, and the data are presented as the mean ± SEM of three independent experiments (∗*p* < 0.05; ∗∗*p* < 0.01). CCCP, carbonyl cyanide 3-chlorophenylhydrazone; CQ, chloroquine; FBW7, F-box and WD repeat domain–containing 7; MEF, mouse embryonic fibroblast; PINK1, PTEN-induced kinase 1.

We then investigated the variations in mitochondrial proteins, including the outer mitochondrial membrane proteins VDAC1, Mfn2, and Tom20, the mitochondrial matrix protein HSP60, and the inner mitochondrial membrane (IMM) protein COX4, aiming to elucidate their potential roles in the observed cellular responses. Firstly, *FBW7*-KO or control HCT116 cells were treated with CCCP in a time-dependent manner to assess the impact on mitochondrial protein levels. Western blot analysis of cell lysates revealed a significant decrease in mitochondrial proteins, including VDAC1, HSP60, COX4, Mfn2, and Tom20, in FBW7^−/−^ cells upon CCCP treatment compared to FBW7^+/+^ cells (Fig. 6, *A* and *B*). Similar experiments were conducted in SH-SY5Y cells treated with *FBW7β*-siRNA, followed by CCCP exposure. The blockade of FBW7β expression led to a substantial accumulation of PINK1 under CCCP treatment, accompanied by a notable reduction in mitochondrial protein levels (Fig. 6, *C* and *D*). Moreover, mouse embryonic fibroblasts (MEFs) derived from *PINK1*-null (PINK1^−/−^) and control (PINK1^+/+^) mice were employed to further investigate these effects. Immunoblotting analysis of PINK1^+/+^ MEFs demonstrated increased PINK1 levels and decreased mitochondrial proteins under *FBW7β*-knockdown plus CCCP treatment, mirroring the results in Figure 6*C*. In contrast, PINK1^−/−^ MEFs showed minimal reduction in mitochondrial proteins under *FBW7β* knockdown and CCCP treatment (Fig. 6, *E* and *F*). Finally, we examined how the changes in these mitochondrial proteins were affected by CQ treatment. As a result, we observed that the levels of those proteins significantly were reduced by CCCP treatment and FBW7β inhibition, whereas they were restored by CQ treatment (Fig. 6, *G* and *H*).

**Figure 7:**
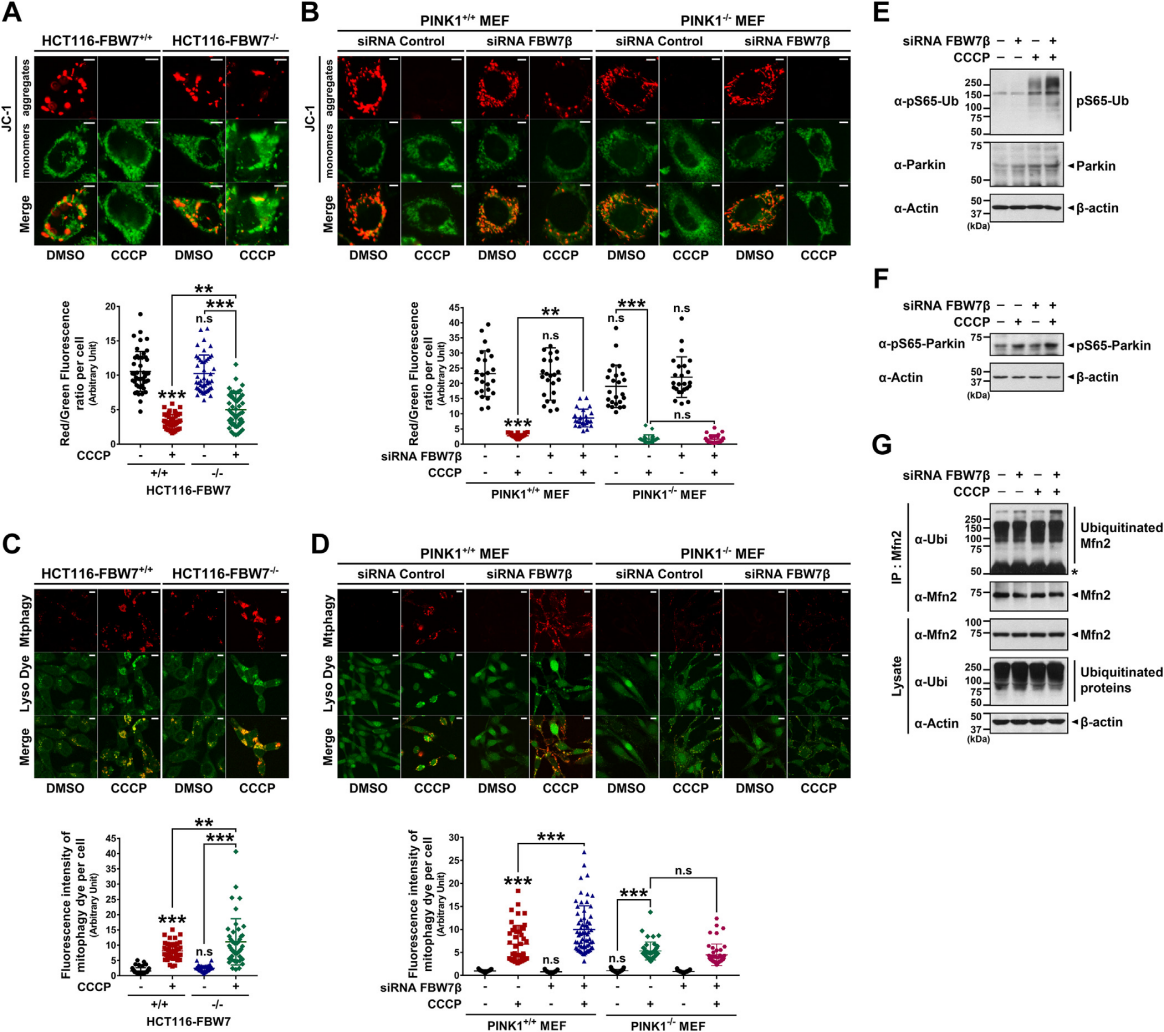
**FBW7β depletion promotes CCCP-induced mitophagy *via* PINK1–Parkin pathway.***A*, FBW7^+/+^ and FBW7^−/−^ HCT116 cells were treated with vehicle or 20 μM CCCP for 24 h. The MMP in each cell was detected using JC-1 staining. Representative confocal images of JC-1 aggregates (*red*) and JC-1 monomers (*green*) are shown. The scale bars represent 5 μm. The MMP (*red*/*green* fluorescence ratio) were quantified using Image J software and depicted as a scatter plot. Data are presented as the mean ± SEM of three independent experiments (n = 45; ∗∗*p* < 0.01; ∗∗∗*p* < 0.001; n.s, not significant). *B*, PINK1^+/+^ and PINK1^−/−^ MEF cells were transfected for 48 h with scrambled control siRNA or *FBW7β*-siRNA and treated with vehicle or 10 μM CCCP for additional 24 h. The MMP of each sample was detected using JC-1 staining. Representative confocal images of JC-1 aggregates (*red*) and monomers (*green*) are shown. The scale bars represent 5 μm. The MMP values were quantified using Image J software and depicted as a scatter plot. Data are presented as the mean ± SEM of three independent experiments (n = 24; ∗∗*p* < 0.01; ∗∗∗*p* < 0.001; n.s, not significant). *C*, FBW7^+/+^ and FBW7^−/−^ HCT116 cells were treated with vehicle or 20 μM CCCP for 24 h. The mitophagy (*red*) and lysosome dyes (*green*) were then used to stain each sample using the mitophagy detection kit (Dojindo). Representative confocal images showing the colocalization of mitophagy (*red*) and lysosome (*green*) are presented. The scale bars represent 10 μm. The extent of mitophagic flux (fluorescence intensity of *red*) were quantified using Image J software and depicted as a *scatter plot*. Data are presented as the mean ± SEM of three independent experiments (n = 45; ∗∗*p* < 0.01; ∗∗∗*p* < 0.001; n.s, not significant). *D*, PINK1^+/+^ and PINK1^−/−^ MEF cells were transfected for 48 h with scrambled control siRNA or *FBW7β*-siRNA and treated with vehicle or 10 μM CCCP for additional 24 h. The mitophagy (*red*) and lysosome dyes (*green*) were then used to stain each sample. Representative confocal images showing the colocalization of mitophagy (*red*) and lysosome (*green*) are presented. The scale bars represent 10 μm. The extent of mitophagic flux (fluorescence intensity of *red*) were quantified using Image J software and depicted as a *scatter plot*. Data are presented as the mean ± SEM of three independent experiments (n = 45; ∗∗∗*p* < 0.001; n.s, not significant). *E*, HEK293 cells were transfected with scrambled control siRNA or *FBW7β*-siRNA for 48 h and treated with vehicle or 10 μM CCCP for additional 24 h. Cell lysates were immunoblotted with the indicated antibodies. β-Actin was used as a control for equal protein loading. *F*, SH-SY5Y cells were transfected with scrambled control siRNA or *FBW7β*-siRNA for 48 h and treated with vehicle or 10 μM CCCP for additional 24 h. Cell lysates were immunoblotted with the indicated antibodies. β-Actin was used as a control for equal protein loading. *G*, HEK293 cells were transfected with scrambled control siRNA or *FBW7β*-siRNA for 48 h and treated with 20 μM MG132 for 30 min prior to addition of CCCP. Cells were then treated with vehicle or 20 μM CCCP for additional 3 h. Cell lysates were immunoprecipitated with anti-Mfn2 antibody, followed by immunoblotting with the indicated antibodies. The *asterisk* indicates IgG heavy chains. β-Actin served as a loading control. CCCP, carbonyl cyanide 3-chlorophenylhydrazone; FBW7, F-box and WD repeat domain–containing 7; MEF, mouse embryonic fibroblast; PINK1, PTEN-induced kinase 1.

We then explored whether FBW7 depletion and the resulting PINK1 accumulation enhance mitophagy. To investigate this, we used JC-1 dye staining, a fluorescent probe indicating mitochondrial membrane potential (MMP), to assess the mitochondrial depolarization in cells treated with vehicle or CCCP. As anticipated, CCCP treatment caused a significant reduction in MMP within FBW7^+/+^ cells (Fig. 7*A*). Notably, FBW7 depletion rescued the CCCP-induced loss of MMP in FBW7^−/−^ HCT116 cells (Fig. 7*A*). Similarly, using PINK1^+/+^ and PINK1^−/−^ MEFs, we evaluated the effect of *FBW7* knockdown on MMP. PINK1^+/+^ MEFs treated with *FBW7β*-siRNA exhibited a significant increase in CCCP-induced MMP loss (groups 2 and 4 in Fig. 7*B*). Moreover, the effect of FBW7 depletion was seen in PINK1^+/+^ MEFs, but not in PINK1^−/−^ MEFs (groups 6 and 8 in Fig. 7*B*). These findings imply a potential correlation between FBW7β and mitochondrial depolarization, suggesting its involvement in the regulation of PINK1-dependent mitophagy.

To verify these results, we further analyzed the effect of FBW7β on mitophagy using a mitophagy detection kit (MD01, Dojindo). This kit, coupled with fluorescence microscopic analysis, allows the observation of an amplified red fluorescence signal due to fusion with lysosomes during mitophagy induction, leading to a decrease in pH. Moreover, an increase in colocalization between damaged mitochondria and lysosomes serves as an additional indicator of mitophagy occurrence. As illustrated in Figure 7*C*, cell staining analysis with mitophagy and lysosome dyes revealed a heightened level of mitophagy in FBW7^−/−^ HCT116 cells compared to FBW7^+/+^ cells upon CCCP treatment as a control. Furthermore, when both PINK1^+/+^ and PINK1^−/−^ MEFs cells were treated with *FBW7β*-siRNA, followed by CCCP exposure, a significant increase in mitophagy was observed in PINK1^+/+^ MEFs. However, this effect was not observed in PINK1^−/−^ MEFs treated with *FBW7β*-siRNA and CCCP (Fig. 7*D*).

In addition, we investigated whether the CCCP-induced mitophagy altered by FBW7 depletion is associated with the PINK1–Parkin pathway. Initially, HEK293 cells were treated with *FBW7β*-siRNA, followed by CCCP treatment, and changes in pSer65-ubiquitin were observed through Western blot analysis. Interestingly, we observed a greater increase in the levels of pSer65-ubiquitin when FBW7β was inhibited along with CCCP treatment, compared to that with CCCP treatment alone (Fig. 7*E*). Subsequently, we examined the phosphorylation of Parkin at Ser65 in SH-SY5Y cells. Similar to the observations in HEK293 cells, we noted a greater increase in the phosphorylation of Parkin at Ser65 when FBW7β was knocked down with CCCP treatment compared to that with CCCP treatment alone (Fig. 7*F*). Lastly, we evaluated the extent of ubiquitination of Mfn2, a well-known substrate of Parkin E3 ligase. Parkin mediates the ubiquitination of various mitochondrial proteins to induce proteasomal degradation when mitophagy occurs (6). As expected, we observed the increase in the ubiquitination of Mfn2 in the lane where FBW7β was knocked down, followed by CCCP treatment (Fig. 7*G*). In summary, inhibiting FBW7β induces the accumulation of PINK1, thereby promoting CCCP-induced mitophagy, and this process is likely mediated through the PINK1–Parkin pathway.

### FBW7β enhances staurosporine-induced cell death *via* PINK1 proteolysis

**Figure 8:**
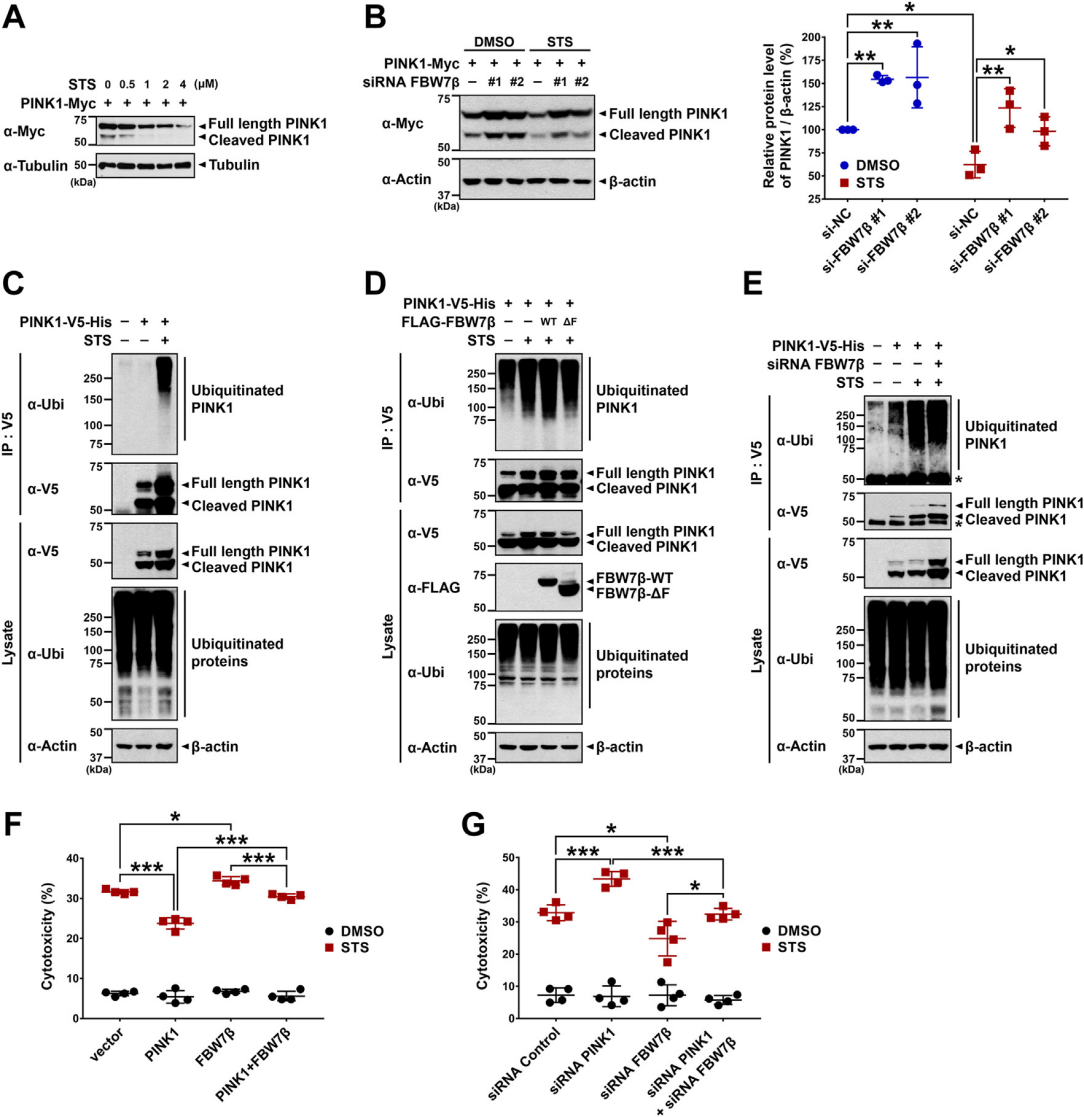
**FBW7β promotes staurosporine-induced cell death via PINK1 proteolysis.***A*, HEK293 cells were transfected with plasmid encoding PINK1-Myc for 24 h and treated with the indicated concentrations of STS for an additional 6 h. Cell lysates were immunoblotted using an anti-Myc antibody. Tubulin served as a loading control. *B*, HEK293 cells were sequentially transfected for 24 h with nonspecific control siRNA or *FBW7β*-siRNA and with PINK1-Myc for additional 24 h. Cells were then treated with either vehicle or 1 μM STS for 6 h. Cell lysates were immunoblotted with anti-Myc antibody. β-Actin served as a loading control. The relative PINK1 levels compared to β-actin were quantified. The presented data represent the mean ± SEM of three independent experiments (∗*p* < 0.05; ∗∗*p* < 0.01). *C*, where indicated, HEK293 cells were mock-transfected or transfected with plasmid encoding PINK1-V5-His for 24 h and treated sequentially with vehicle or 1 μM STS for 8 h and with 20 μM MG132 for additional 4 h. Cell lysates were immunoprecipitated with anti-V5 antibody, followed by immunoblotting with the specified antibodies. β-Actin served as a loading control. *D*, HEK293 cells were transfected for 24 h with plasmids encoding PINK1-V5-His, FLAG-FBW7β-WT, or FLAG-FBW7β-ΔF alone or in combination. Cells were then treated with vehicle or 1 μM STS for 8 h and treated with 20 μM MG132 for additional 4 h. Cell lysates were immunoprecipitated with anti-V5 antibody, followed by immunoblotting with the specified antibodies. β-Actin served as a loading control. *E*, HEK293 cells were sequentially transfected for 24 h with nonspecific control siRNA or *FBW7β*-siRNA and with PINK1-V5-His for additional 24 h. Cells were then treated with vehicle or 1 μM STS for 8 h and with 20 μM MG132 for additional 4 h. Cell lysates were immunoprecipitated with anti-V5 antibody, followed by immunoblotting with the specified antibodies. The *asterisk* indicates IgG heavy chains. β-Actin served as a loading control. *F*, SH-SY5Y cells were either mock-transfected or transfected with plasmids encoding Myc-PINK1 or/and FLAG-FBW7β for 24 h and treated with vehicle or 1 μM STS for an additional 24 h. Cell toxicity was measured using an LDH assay kit. The data are presented as the mean ± S.D. of four independent experiments (n = 4; ∗, *p* < 0.05; ∗∗∗, *p* < 0.001). *G*, SH-SY5Y cells were transfected with nonspecific control siRNA, *FBW7β*-siRNA, or *PINK1*-siRNA alone or in combination for 48 h and treated with vehicle or 1 μM STS for an additional 24 h. Cell toxicity was measured using an LDH assay kit. The data are presented as the mean ± S.D. of four independent experiments (n = 4; ∗, *p* < 0.05; ∗∗∗, *p* < 0.001). FBW7, F-box and WD repeat domain–containing 7; LDH, lactate dehydrogenase; PINK1, PTEN-induced kinase 1; PLA, proximity ligation assay; STS, staurosporine.

Finally, we investigated whether FBW7β and the consequent suppression of mitophagic progression *via* PINK1 proteolysis affects cell viability. In a prior study, we demonstrated that CHIP-mediated degradation of PINK1 intensifies cell death induced by exposure to toxic staurosporine (STS) (9). In this context, we aimed to verify whether FBW7β, akin to CHIP E3 ligase, facilitates the degradation of neuroprotective PINK1, thereby further promoting cell demise induced by STS. Initially, we observed a gradual decrease in PINK1 levels with escalating concentrations of STS in HEK293 cells transfected with PINK1-Myc as a control (Fig. 8*A*), aligning with previous findings (9). Subsequently, when HEK293 cells were treated with *FBW7β*-siRNA, followed by vehicle or STS, the siRNA-mediated downregulation of FBW7β inhibited PINK1 degradation, even under STS treatment, maintaining elevated PINK1 levels (Fig. 8*B*). Coinciding with increased cell death, STS treatment correspondingly elevated PINK1 ubiquitination levels (Fig. 8*C*). However, this STS-induced stimulation of PINK1 ubiquitination was significantly diminished by an FBW7β-ΔF mutant (Fig. 8*D*). Additionally, siRNA-mediated knockdown of endogenous FBW7β inhibited the increased PINK1 ubiquitination induced by STS (Fig. 8*E*). These findings imply the role of FBW7β in negatively regulating PINK1 stability, thereby enhancing cell demise. To further substantiate this hypothesis, we conducted lactate dehydrogenase (LDH) assays to evaluate the cytotoxic effects of FBW7β and PINK1 degradation in SH-SY5Y cells. The LDH assay confirmed that FBW7β-mediated PINK1 degradation was further intensified upon STS treatment, accompanied by an increase in cytotoxicity (Figs. 8, *F* and *G*, and S1*C*).

In conclusion, the overall data support the notion that FBW7β promotes STS-induced cell death through PINK1 proteolysis.

## Discussion

While the crucial role of PINK1 in various physiological processes is well understood, the regulatory pathway governing PINK1 stability has not been fully elucidated. Previous studies have demonstrated that lysosomal inhibitors have minimal impact on PINK1, whereas proteasome inhibitors effectively hinder its degradation (28). Although the precise mechanism controlling PINK1 still need further elucidation, these findings strongly indicate that the homeostatic level of PINK1 is primarily maintained through UPS, not the autophagy pathway. This speculation finds support in our current study, where the use of MG132 or epoxomicin confirmed a significant inhibition of PINK1 degradation (Fig. 3*A*). Integrating these results with prior research (9, 28, 29), it can be concluded that the regulation of PINK1 degradation predominantly occurs through the UPS pathway. Consistent with these ideas, two enzymes involved in protein ubiquitination, UBR and CHIP, have been identified, both promoting the proteolytic degradation of PINK1 through the proteasome pathway (8, 9, 30).

Under normal mitochondrial conditions, PINK1 undergoes rapid and continuous degradation. Its N terminus is inserted into the IMM through the translocase of the outer membrane (TOM) and TIMM23 translocator complexes (31, 32). Within the IMM, an IMM protease called PARL cleaves PINK1 between Ala-103 and Phe-104 residues, releasing a C-terminal fragment (8, 30, 31, 32, 33). This fragment is subsequently recognized by UBR1, UBR2, and UBR4 and is eliminated through UPS (8, 32). Additionally, the 52 kDa form of PINK1 is present in both mitochondrial and endoplasmic reticulum (ER)-enriched fractions in steady state. It interacts with proteins from the ER-associated degradation pathway, including ERAD E3 ligases gp78 and HRD1, which collaborate to promote the ubiquitination of PINK1, leading to proteasomal degradation (34, 35). Furthermore, our previous findings indicated that CHIP functions as a novel ubiquitin E3 ligase responsible for targeting full-length PINK1, resulting in its ubiquitination and subsequent degradation *via* the proteasome pathway (9). The present study uncovers another member in the category of PINK1-targeting E3 ligases, FBW7β, which facilitates the ubiquitination of PINK1, thereby marking it for UPS-mediated degradation.

Several proteins have been identified to influence the stability of PINK1. Notably, members of the Bcl-2-associated athanogene (BAG) family, specifically BAG2 and BAG5, have been found to stabilize PINK1 by reducing its ubiquitination (36, 37). Additionally, DJ-1, an autosomal recessive early-onset familial gene associated with PD, directly interacts with PINK1 and elevates its cellular levels (28). The increase in HtrA2, another PD-specific mutation gene member, is known to correlate with elevated PINK1 levels, establishing a connection between these two proteins (38).

Apart from proteolytic regulation, some factors affect the mitochondrial processing of PINK1. For instance, the human telomerase reverse transcriptase (hTERT) has been shown to increase mitophagy by impeding PINK1 processing, leading to the accumulation of full-length PINK1 on the outer mitochondrial membrane (39). Conversely, Tollip promotes PINK1 processing within mitochondria, releasing cleaved PINK1 into the cytosol and inhibiting mitophagy (40). Various posttranslational modifications, such as phosphorylation, also impact PINK1. Kinases like AMPKα2 and MARK2 can phosphorylate PINK1, with AMPKα2 phosphorylating PINK1 at Ser-495, enhancing mitophagy and mitigating mitochondrial dysfunction (41). MARK2 phosphorylates PINK1 at Thr-313, influencing its mitochondrial transport and function in neurons (42). Additionally, NEDD8 conjugation to PINK1 selectively stabilizes the 55 kDa PINK1 fragment, maintaining its cleavage product within cells (43). The existence of these regulatory reactions, including the newly discovered mode involving FBW7β, underscores the functional significance of PINK1 in regulating mitochondrial dynamics and apoptosis, enabling rapid adjustments in response to diverse environments.

The SCF–FBW7 complex functions as a multisubunit RING-finger E3 ligase, targeting a variety of proteins for degradation (10, 11, 12). Its established role in downregulating oncogenic proteins underscores its importance in tumor suppression (10, 11, 12). FBW7, a key player in this complex, selectively identifies substrate proteins and directs them for ubiquitination, culminating in their subsequent proteasomal degradation (13, 14, 15). Dysfunctions in FBW7 or disruptions in FBW7-mediated protein degradation pathways have also been linked to neurodegenerative diseases, including AD and PD (23). These dysfunctions can lead to abnormal protein accumulation and nerve cell degeneration, underscoring the pivotal role of FBW7 in the onset of these disorders (23).

In the context of AD, FBW7 interacts with presenilin 1, a vital component of γ-secretase responsible for cleaving amyloid precursor protein into amyloid-β. This interaction promotes presenilin 1 ubiquitination, potentially regulating amyloid-β generation in AD (44). Additionally, FBW7 modulates neuronal apoptosis in AD by mediating the proteolysis of c-Jun (45, 46) and cyclin E1 (47), essential factors in promoting neuronal apoptosis (48, 49). Moreover, FBW7 might mitigate neuronal apoptosis through the proteasome-dependent degradation of RCAN1 (50), elevated in AD patient brains and implicated in neuronal apoptosis (51, 52). However, there are contradictory reports suggesting that FBW7β promotes neuronal apoptosis by mediating the ubiquitination-dependent proteolysis of Mcl-1 (53). These findings indicate that FBW7β can exhibit either cellular protective or cytotoxic activity, depending on cellular context and substrates. This study further adds evidence to FBW7β′s cytotoxic action, transmitting STS toxicity *via* PINK1 degradation.

Regarding its connection to PD, FBW7β levels were considerably elevated in the cortexes of PD patients with Parkin gene mutations. This study suggests that dysfunctional Parkin resulted in the inhibition of FBW7β ubiquitination and subsequent proteasome degradation (53). Conversely, another study showed that FBW7β stability was decreased in response to 6-hydroxydopamine, a neurotoxic inducer of PD pathology in animal models. This study proposed that 6-hydroxydopamine induced the oxidation of FBW7β, leading to its binding with HSP70, a crucial regulator of chaperone-mediated autophagy, and subsequent degradation of FBW7β through chaperone-mediated autophagy (54). The present study provides further evidence of a close link between PD and FBW7β. Here, we demonstrate that FBW7β mediates the ubiquitination of PD-linked PINK1, leading to its proteasomal degradation and exacerbating STS-mediated cell death. Based on the well-established finding that Parkin is recruited when PINK1 accumulates under mitophagic stress, it can be speculated that depletion of FBW7β may lead to an increase in Parkin levels. This hypothesis was supported by a previous discovery that Parkin mediates the proteasomal degradation of FBW7β (53), possibly contributing to maintaining cellular homeostasis in the mitophagy process. While there have not been any reports clarifying the role of FBW7 in controlling mitochondrial function, one of the main causes of PD, this study provides the first evidence that FBW7β affects mitochondrial dynamics through the regulation of PINK1.

In summary, this study uncovers the role of FBW7β as a novel E3 ligase for PINK1, elucidating its involvement in the degradation of PINK1 and subsequent reduction in cellular protective function. Consequently, FBW7β negatively affects CCCP-induced mitophagy and promotes STS-induced cell death. Furthermore, the implications of FBW7β in neurological disorders and the potential therapeutic significance of PINK1 in cancer highlight the broader implications of this research.

## Experimental procedures

### Materials

Dulbecco's modified Eagle medium (DMEM) and fetal bovine serum (FBS) were purchased from Corning Life Science. Lipofectamine 2000 was purchased from Invitrogen. Protein A-Sepharose beads were obtained from GE Healthcare Life Sciences. Protein G-agarose 4B resin was obtained from Lugen Sci. Enhanced chemiluminescence reagents were obtained from PerkinElmer Life and Analytical Sciences, AbClon, and Advansta. MG132 was provided from AG Scientific. STS (S4400) and cycloheximide (C4859) were obtained from Sigma-Aldrich. The monoclonal anti-V5 antibody (46-0705) was purchased from Invitrogen. Monoclonal anti-FLAG (F3165) antibodies were obtained from Sigma-Aldrich. Monoclonal anti-HSP90 (sc-13119), monoclonal anti-β-actin (sc-47778), monoclonal anti-Myc (sc-40), and monoclonal anti-ubiquitin (sc-8017) were purchased from Santa Cruz Biotechnology (Santa Cruz). Monoclonal anti-hemagglutinin antibody (MMS-101P) was obtained from Covance. Polyclonal rabbit anti-PINK1 (BC100-494) antibody was obtained from Novus Biologicals. Polyclonal rabbit anti-FBW7β antibody (H00055294-D01P) was purchased from Abnova. Polyclonal rabbit anti-FBW7 IgG (A301-720A) was purchased form Bethyl Laboratories. Monoclonal anti-tubulin (GTX628802) antibody was purchased from Genetex. Polyclonal anti-V5 (ab9116) antibody was obtained from Abcam. Polyclonal anti-pSer65-Parkin (#36866) antibody was obtained from Cell Signaling Technology. Polyclonal anti-pSer65-ubiquitin (ABS1513-I) antibody, mouse IgG (12–371), rabbit IgG (12–370), peroxidase-conjugated mouse IgG (AP124P), and peroxidase-conjugated rabbit IgG (AP132P) were obtained from Millipore. Alexa Fluor 488–conjugated anti-mouse (A-11029) and Alexa Fluor 594–conjugated anti-rabbit (A-11012) secondary antibodies were purchased from Invitrogen. Analytical grade commercial products obtained from Sigma-Aldrich were used as the source of all other chemicals in this study.

### DNA constructs

The mammalian construct encoding Myc-tagged human WT PINK1 (pBOS-hPINK1-WT-3X-Myc) was generously provided by Dr J. Chung (Seoul National University). Plasmids encoding FLAG-tagged FBW7α, FBW7β, and FBW7γ were kindly provided by Dr B.E. Clurman (Fred Hutchinson Cancer Center). The plasmid encoding Myc-tagged PINK1 was subcloned into a pcDNA-V5-His vector (PINK1-V5-His). The plasmid encoding FLAG-tagged FBW7β-WT was deleted to construct an FBW7β mutant lacking the F-box domain (FBW7β-ΔF). All DNA sequences were confirmed by sequencing (BIONICS).

### Cell culture and DNA transfection

Human colon cancer HCT116 FBW7^+/+^ and FBW7^−/−^ cells were kindly provided from Drs B. Vogelstein (Johns Hopkins University School of Medicine) and Y.Y. Cho (The Catholic University of Korea). MEFs derived from *PINK1-*null (PINK1^−/−^) and control (PINK1^+/+^) mice were provided by J. Shen (Harvard Medical School). HEK293 cells, PINK1 MEFs, and HCT116-FBW7 cells were maintained in DMEM supplemented with 10% FBS and 100 U/ml penicillin–streptomycin. Human neuroblastoma SH-SY5Y cells were maintained in 1:1 mixture of DMEM and F12 medium supplemented with 10% FBS and 100 U/ml penicillin–streptomycin. The cells were grown at 37 °C in a humidified atmosphere containing 5% CO_2_. All DNA transfections were performed using Lipofectamine 2000, according to the manufacturer’s protocol. To induce mitophagy, cells were treated with CCCP for the indicated times and concentrations. To inhibit autophagy, cells were treated with CQ for the indicated times and concentrations. To induce apoptotic cell death, cells were treated with STS for the indicated times and concentrations.

### RNA interference

The siRNAs targeting *FBW7β*, *PINK,1* or *cullin-1* were synthesized by Bioneer. Scrambled siRNA (catalog no.: # 51-01-14-04) as a negative control was purchased from integrated DNA technologies. The *FBW7β*-specific siRNA #1 duplex sense and antisense sequences were 5′-UAUGGGUUUCUACGGCACAdTdT-3′ and 5′-UGUGCCGUAGAAACCCAUAdTdT-3′, respectively. The *FBW7β*-specific siRNA #2 duplex sense and antisense sequences were 5′- ACAGGACAGUGUUUACAAAdTdT-3′ and 5′-UUUGUAAACACUGUCCUGUdTdT-3′, respectively. The *PINK1*-specific siRNA duplex sense and antisense sequences were 5′- GAAAUCCGACAACAUCCUUUUdTdT-3′ and 5′-AAAAGGAUGUUGUCGGAUUUCdTdT-3′, respectively. The *cullin-1*–specific siRNA #1 duplex sense and antisense sequences were 5′- GCUCUACACUCAUGUUUAUdTdT-3′ and 5′-AUAAACAUGAGUGUAGAGCdTdT-3′, respectively. The *cullin-1*–specific siRNA #2 duplex sense and antisense sequences were 5′- GACGAAGGACGAAAAGGAAdTdT-3′ and 5′-UUCCUUUUCGUCCUUCGUCdTdT-3′, respectively. The *cullin-1*–specific siRNA #3 duplex sense and antisense sequences were 5′- CAUUUUGGCGCAAGUUUUAdTdT-3′ and 5′-UAAAACUUGCGCCAAAAUGdTdT-3′, respectively. The *cullin-1*–specific siRNA #4 duplex sense and antisense sequences were 5′- CUAAACUUCAGCGCAUGUUdTdT-3′ and 5′-AACAUGCGCUGAAGUUUAGdTdT-3′, respectively.

### Immunoprecipitation and western blotting analysis

Cultured cells were harvested by scraping with ice-cold PBS, washed with PBS, and incubated at 4 °C for 30 min with lysis buffer containing 50 mM Tris (pH 7.5), 150 mM NaCl, 1.0% Nonidet P-40, and 10% glycerol. Aliquots of protease inhibitor cocktail including 0.2 mM PMSF, 1 μg/ml aprotinin, 1 μg/ml leupeptin, 1 mM Na_3_VO_4_, and 10 mM NaF were added to cell lysates just before use. Cell lysates were then vortexed every 10 min, centrifuged at 15,000*g* for 15 min at 4 °C, and the supernatants were collected into clean tubes. For immunoprecipitation, cell lysates including 0.5∼2 mg protein were incubated with the appropriate antibody overnight at 4 °C with gentle rotation. The samples were incubated with either protein A-Sepharose or protein G-Agarose beads, incubated for 2 h at 4 °C, centrifuged at 9300*g* for 30 s, and washed three times with lysis buffer. Immunocomplexes were collected by centrifugation at 8000*g* for 1 min, and bead-bound proteins were eluted by adding 2× SDS-PAGE sample buffer. The samples were denatured by boiling for 5 min, separated on SDS-PAGE gels, and transferred onto nitrocellulose membranes. Membranes were blocked for 1 h at room temperature (RT) with 5% nonfat dry milk in 1× Tris-buffered saline with Tween 20 (TBST) buffer [25 mM Tris (pH 7.6), 137 mM NaCl, and 0.1% Tween 20] and incubated with the appropriate primary antibody overnight at 4 °C. Membranes were then washed with TBST, incubated for 2 h with horseradish peroxidase–conjugated secondary IgG, washed again with TBST, and visualized using enhanced chemiluminescence reagents following the manufacturer’s instructions.

### Immunocytochemistry analysis

Cell were cultured on poly-L-lysine-coated glass coverslips in 6-well plates, harvested, washed twice with PBS buffer, and fixed in 3.7% formaldehyde for 10 min at RT. Cells were then permeabilized with 0.2% Triton X-100 for 10 min, blocked with 1% bovine serum albumin (BSA) in PBS for 1 h at RT, and stained with indicated antibodies for 16 h at 4 °C. The samples were incubated with Alexa Fluor 488–conjugated anti-mouse or Alexa Fluor 594–conjugated anti-rabbit secondary antibodies to detect the primary antibodies. Images were captured using an LSM 880 laser scanning confocal microscope (Carl Zeiss), and the data were processed using Zeiss LSM Image Browser (Carl Zeiss) (https://www.zeiss.com/microscopy/en/products/software/light-microscopy-software.html).

### Proximity ligation assay

The proximity ligation assay (PLA) was conducted using the Duolink PLA assay kit (DUO92101, Sigma-Aldrich), following the manufacturer’s protocols. The PLA signals are shown in red, and the nuclei are displayed in blue.

### Mitophagy assay

The mitophagy of cells was analyzed using the Mitophagy detection kit (Dojindo Laboratories), according to the manufacturer’s instructions. Briefly, cultured cells were seeded in μ-Dish 35 mm (Cat. 80136, ibidi GmbH) at a density of 5 × 10^4^ cells per well. The level of mitophagy was detected in red. The levels of lysosomes were shown in green. To quantify the mitophagy signals, Image J software (National Institutes of Health, https://imagej.net/ij/) was used for analysis.

### Analysis of MMP

The MMP (ΔΨm) of cells was analyzed using JC-1 dye (Life Technologies), according to the manufacturer’s instructions. Briefly, cells were stained with 0.5 μg/ml JC-1 for 30 min at 37 °C in 5% CO_2_. Cells were then washed three times with DMEM and analyzed using LSM 880 confocal microscope (Carl Zeiss). The orange- and green-fluorescent signal indicated the cells with hyperpolarized and depolarized membrane potential, respectively. To quantify the ΔΨm, Image J software was used for analysis.

### LDH cytotoxicity assay

Cellular toxicity was measured using an LDH cytotoxicity assay kit (Cat. MK401, Takara), according to the manufacturer’s instructions. Briefly, after DNA or siRNA transfection for 48 h, cells were either left untreated or treated with 1 μM STS for an additional 24 h. Cell-free culture media were then transferred to sterile 96-well plates, and the appropriate mixture of kit solutions was added to each well. The plates were incubated for up to 30 min at RT to determine the LDH activity in the supernatant. The absorbance of each sample was measured at 490 to 492 nm using a microplate reader.

### Statistical analysis

One-way ANOVA with unpaired *t* tests was used for all statistical analyses to compare data from different groups. The analysis was performed using SPSS statistical analysis software (version 25.0; IBM, https://www.ibm.com/kr-ko/spss). All values are reported as mean ± SEM; *p* values less than 0.05 were considered statistically significant, and the sample size for each experiment (n) is noted in the corresponding Figure legends. The intensities of Western blot bands were measured using GelQuant.NET software (version 1.8.2; biochemlabsolutions.com).

## Data availability

All datasets are included within the manuscript or are available from the corresponding author: Kwang Chul Chung (kchung@yonsei.ac.kr).

## Supporting information

This article contains Supporting information.

## Conflict of interest

The authors declare that they have no conflicts of interest with the contents of this article.
