# Supplementary material for: The SCF-FBW7β E3 ligase mediates ubiquitination and degradation of the serine/threonine protein kinase PINK1
Source: J Biol Chem. 2024 Mar 18;300(4):107198. doi: 10.1016/j.jbc.2024.107198 (PMC11026729; doi:10.1016/j.jbc.2024.107198)
Supplement: Supporting Figure S1 [file mmc1.pdf]

Supporting information

The SCF-FBW7β E3 ligase mediates ubiquitination and degradation of the serine/threonine protein kinase PINK1

Seo Jeong Jeon and Kwang Chul Chung

Contents

Figure S1. Real-time quantitative PCR analyses for verifying knockdown and knockout effects.

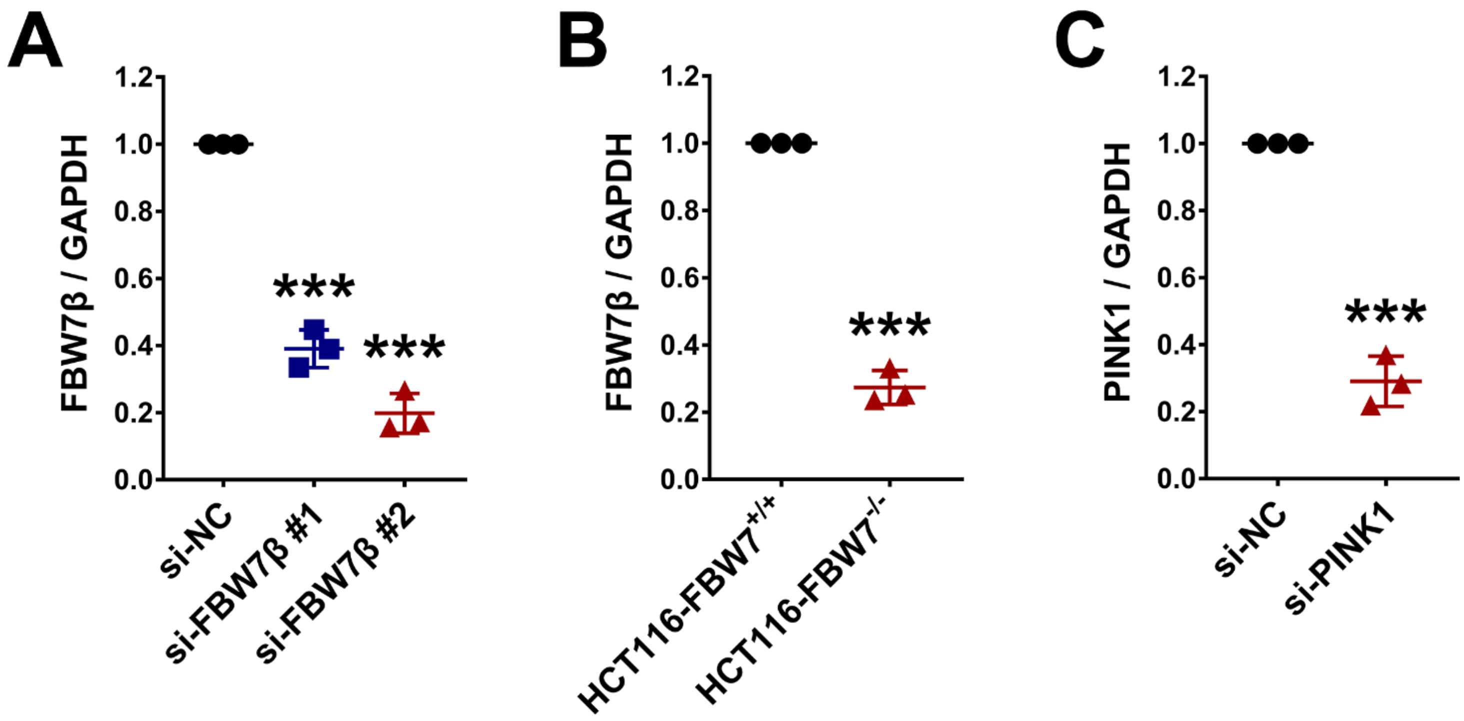

Figure S1. Verification of knockdown and knockout effects using real-time quantitative PCR. (A) HEK293 cells were transfected with nonspecific control siRNA or *FBW7β*-siRNA for 48 h. Total RNA was extracted and reverse transcribed. Relative *FBW7β* mRNA levels were measured using real-time PCR. Two different siRNAs targeting *FBW7β* were used. The data are presented as the mean ± S.D. of three independent experiments (n = 3; ***, $p < 0.001$). **(B)** $FBW7^{+/+}$ and $FBW7^{-/-}$ HCT116 cells were harvested after 48 h cultured. Total RNA was extracted and reverse transcribed. Relative *FBW7β* mRNA levels were measured using real-time PCR. The data are presented as the mean ± S.D. of three independent experiments (n = 3; ***, $p < 0.001$). **(C)** SH-SY5Y cells were transfected with nonspecific control siRNA or *PINK1*-siRNA for 48 h. Total RNA was extracted and reverse transcribed. Relative *PINK1* mRNA levels were measured using real-time PCR. The data are presented as the mean ± S.D. of three independent experiments (n = 3; ***, p < 0.001). All relative mRNA levels were normalized to GAPDH.
